# 

**Published:** 1883-07

**Authors:** 


Whole Number, CCLIV. JULY, 1883. Number 12, Volume XXII.

### THE BUFFALO Medical and Surgical Journal.

INDEXED

EDITORS:

BUFFALO:

Published by the Medical Journal Association.

No. 5 WEST CHIPPEWA ST.

**Read the Notice of this Journal among the Advertisements.**

Entered at the Post-Office at Buffalo, N. Y., as Second-class Mail Matter.

